# Sudden death across brain and heart: cardio-cerebral ion channel dysfunction as a potential risk-modifying mechanism linking epilepsy and long QT syndrome

**DOI:** 10.3389/fneur.2026.1857624

**Published:** 2026-06-05

**Authors:** Jingxiang Zhang, Qing Chen, Zhijian Ma, Kai Luo, Xinyu Ben

**Affiliations:** 1 Department of Neurology, Hainan General Hospital, Hainan Affiliated Hospital of Hainan Medical University & Key Laboratory of Brain Science Research and Transformation in Tropical Environment of Hainan Province, Haikou, Hainan, China; 2 Department of Neurosurgery, Hainan Hospital Affiliated to Hainan Medical University, Haikou, Hainan, China

**Keywords:** cardio-cerebral comorbidity, ion channelopathy, long QT syndrome, precision medicine, sudden unexpected death in epilepsy

## Abstract

Sudden unexpected death in epilepsy (SUDEP) and long QT syndrome (LQTS) are severe disorders causing sudden death in the neurological and cardiovascular systems, respectively, and have traditionally been viewed as distinct clinical entities. However, their overlapping clinical phenotypes and partially shared genetic backgrounds suggest that, in a subset of patients, overlapping molecular mechanisms may contribute to neurocardiac vulnerability. This review systematically elucidates and bridges the potential mechanistic intersections between these two conditions at the level of ion channel dysfunction. It focuses on key shared ion channel genes, such as *KCNQ1*, *KCNH2*, and *SCN5A*, and analyzes how their variants may simultaneously induce neuronal hyperexcitability and cardiomyocyte repolarization abnormalities, thereby suggesting a potential risk-modifying framework of “cardio-cerebral ion channelopathies” in specific clinical scenarios. Additionally, this article discusses the significant implications of this shared mechanism for clinical practice, particularly in evaluating the cardiac safety of anti-seizure medications (ASMs), integrating genetic risk stratification, and developing future therapeutic strategies. By synthesizing and integrating existing evidence, this review aims to provide a broader pathophysiological perspective on the intrinsic links between SUDEP and LQTS, offering novel insights for interdisciplinary precision medicine.

## 1 Introduction

Sudden unexpected death in epilepsy (SUDEP) is one of the leading fatal complications among epilepsy patients, posing a severe life-threatening risk particularly to those with drug-resistant epilepsy. However, its pathophysiological mechanisms remain incompletely understood, limiting effective prevention and intervention strategies (1–7). Meanwhile, long QT syndrome (LQTS), a hereditary cardiac ion channelopathy, is a major cause of sudden cardiac death (SCD) in adolescents and young adults (8–16). Traditionally, SUDEP and LQTS have been regarded as independent clinical entities, belonging to neurological and cardiovascular disorders, respectively.

Nevertheless, accumulating clinical and genetic evidence challenges this conventional classification, suggesting that overlapping pathophysiological mechanisms may exist in selected patients. Epidemiological studies indicate that QT interval prolongation on electrocardiograms (ECGs) in epilepsy patients is an independent predictor of all-cause mortality, revealing for the first time a clinical association between cardiac repolarization abnormalities and adverse outcomes in epilepsy (17). More direct evidence comes from molecular genetics research, where systematic postmortem genetic analyses have frequently identified variants previously thought to be associated only with primary arrhythmias, particularly in genes encoding cardiac ion channels, highlighting a significant genetic overlap between SUDEP and SCD (18, 19).

**Figure 1:**
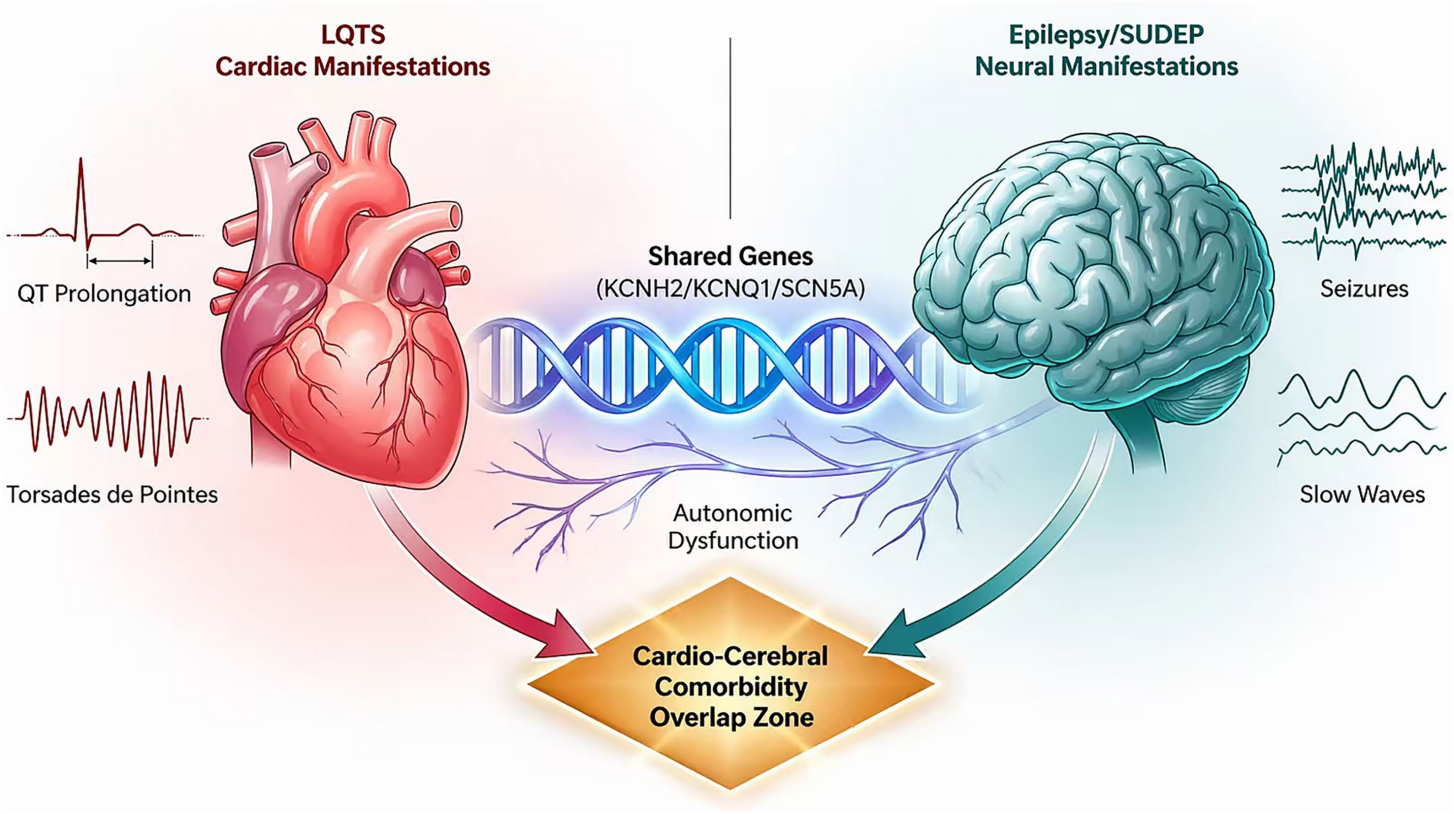
Conceptual framework of the cardio-cerebral channelopathy linking long QT syndrome (LQTS) and epilepsy/SUDEP. This schematic illustrates the profound phenotypic and genetic intersection between LQTS and epilepsy/sudden unexpected death in epilepsy (SUDEP), supporting the concept that shared ion channel dysfunction may contribute to neurocardiac vulnerability in a subset of patients. The left panel depicts typical cardiac manifestations of LQTS, including QT prolongation and Torsades de Pointes. The right panel shows neurological features of epilepsy/SUDEP, such as seizures, EEG slowing, and autonomic dysfunction. The overlapping region represents the core convergence characterized by clinical comorbidity and a shared molecular basis driven by pleiotropic genes (e.g., *KCNH2*, *KCNQ1*, *SCN5A*). Variants in these genes simultaneously disrupt electrophysiological homeostasis in both cardiomyocytes and neurons, highlighting the critical pathophysiological link between disorders traditionally categorized into distinct cardiac and neurological domains.

In light of this, this article proposes an integrative hypothesis: for some patients, SUDEP and LQTS may share a potential risk-modifying foundation, which we term “cardio-cerebral ion channelopathies,” rather than representing isolated disease entities. Centering on this core argument, the review first systematically examines the intersections in their clinical phenotypes and genetic backgrounds, then delves into the “two-hit” model—how genetic susceptibility as an underlying substrate may interact with seizure-related acute physiological stressors, including respiratory dysfunction, hypoxemia, autonomic instability, and acid–base imbalance, to increase vulnerability to fatal cardiorespiratory collapse. Finally, it addresses the profound implications of this integrative perspective for clinical risk assessment, precision diagnosis, and future research directions (Figure 1).

## 2 Clinical phenotypic overlap and epidemiological associations

### 2.1 Neurological manifestations in LQTS and diagnostic challenges

Long QT syndrome (LQTS) and epilepsy exhibit significant phenotypic overlap clinically, posing challenges to differential diagnosis (17, 20–25). In LQTS patients, particularly those with LQT2, cardiac syncope can be accompanied by convulsive activity, making the clinical presentation indistinguishable from epileptic seizures. This similarity often leads to misdiagnosis, resulting in unnecessary anti-seizure medication therapy while the underlying risk of lethal arrhythmias remains unrecognized and unmanaged (23, 24, 26).

Further research suggests that neurological abnormalities in LQTS may not solely be secondary to cerebral hypoperfusion. Electroencephalograms (EEGs) in some LQTS patients show nonspecific slow waves or theta activity, indicating potential primary cortical dysfunction rather than merely hypoxic effects from cardiac events (20, 27, 28). Notably, a history of epilepsy has been identified as an independent risk factor for predicting future fatal cardiac events in diagnosed LQTS cohorts, suggesting a direct pathophysiological link between the two conditions (29).

### 2.2 Cardiac electrophysiological abnormalities in epilepsy patients and SUDEP risk

Correspondingly, cardiac electrophysiological abnormalities are observed in epilepsy populations. Large-scale retrospective studies have confirmed that QT interval prolongation on ECGs in epilepsy patients is an independent predictor of all-cause mortality, indicating that cardiac repolarization abnormalities serve as a key risk marker in this group (17).

This cardio-cerebral association is even more pronounced in SUDEP cases. Autopsy studies reveal that some SUDEP decedents had undiagnosed hereditary cardiac conditions, such as LQTS or Brugada syndrome (30, 31). This link is further substantiated at the molecular level through postmortem genetic analysis (i.e., “molecular autopsy”). Research shows that a substantial proportion of SUDEP cases carry known arrhythmogenic gene variants, which highly overlap with those causative for primary cardiac ion channelopathies (18, 30). In summary, these clinical and epidemiological observations collectively support the possibility of partially overlapping genetic susceptibility in a subset of patients, providing a basis for subsequent in-depth exploration of the molecular mechanisms.

## 3 Shared genetic and molecular mechanisms

The comorbidity between epilepsy and primary arrhythmia syndromes, particularly in assessing SUDEP risk, increasingly points to shared genetic susceptibilities and molecular pathological pathways. Substantial evidence indicates that functional abnormalities in genes encoding key ion channels can simultaneously disrupt electrophysiological homeostasis in brain neurons and cardiac myocytes, potentially contributing to a subset of cases within a “cardio-cerebral channelopathy” risk spectrum. This section aims to systematically elucidate the core genes and signaling pathways linking these two diseases, integrating causal evidence from experimental models (Table 1).

**Table 1:** Key genes and molecular mechanisms linking epilepsy/SUDEP and cardiac arrhythmias in cardio-cerebral channelopathies.

Gene	Encoded protein	Cardiac function	Neuronal function	Associated phenotypes	Representative variants	Mechanism of dual pathophysiology	References
*KCNH2*	K_v_11.1 (hERG)	Mediates rapid delayed rectifier K^+^ current (I_Kr_*I*_*Kr*_); critical for repolarization.	Regulates neuronal excitability and firing frequency.	LQT2, Epilepsy, SUDEP	I82T, R863X, P926AfsX14	Loss-of-function causes prolongation of cardiac action potential duration (APD) and concurrent neuronal hyperexcitability.	(2, 25, 29, 35–37, 40)
*KCNQ1*	K_v_7.1	Mediates slow delayed rectifier K^+^ current (I_Ks_*I_Ks_*); maintains repolarization reserve.	Maintains neuronal electrophysiological stability.	LQT1, Epilepsy, SUDEP	L273F, Q530X	Impaired repolarization leading to phenotypic heterogeneity; cardiac syncope often clinically misdiagnosed as epilepsy due to cerebral hypoperfusion.	(24, 41, 42)
*SCN5A*	Na_v_1.5	Mediates Na^+^ current (I_Na_*I_Na_*); responsible for depolarization and conduction.	Modulates excitability in the limbic system.	LQT3, Brugada Syndrome, Epilepsy, SUDEP	R523C, W1095X	Dysfunction simultaneously compromises cardiac conduction stability and neuronal firing properties.	(30, 31, 43)
*KCND3*	K_v_4.3	Mediates transient outward K^+^ current (I_to_*I_to_*).	Not fully characterized in this context.	ERS, Atrial Fibrillation, Epilepsy, SUDEP	V392I	Complex mixed electrophysiological phenotype resulting in rate-dependent symptoms in both heart and brain.	(44, 45)
*RyR2*	Ryanodine Receptor 2	Controls Ca^2+^ release from sarcoplasmic reticulum.	Maintains Ca^2+^ homeostasis in hippocampus.	CPVT, Epilepsy, SUDEP	R2474S	Diastolic Ca^2+^ leak triggers both cardiac electrical instability (arrhythmia) and neuronal hyperexcitability (seizures).	(46)

### 3.1 Core genes involved in cardio-cerebral ion channelopathies

The molecular association between SUDEP and LQTS is supported by key genes. Pathogenic variants in genes encoding core ion channels, such as *KCNH2*, *KCNQ1*, and *SCN5A*, serve as important genetic risk factors mediating electrophysiological abnormalities in both the heart and brain (32–36). These variants may act as susceptibility factors in a subset of patients, rather than serving as the universal genetic foundation.

#### 3.1.1 *KCNH2* (K_v_11.1)

The *KCNH2* gene encodes the *α*-subunit of the K_v_11.1 (hERG) potassium channel, which plays a critical role in cardiomyocyte repolarization and neuronal excitability regulation in the central nervous system (37). Thus, *KCNH2* dysfunction exemplifies a monogenic defect leading to dual cardio-cerebral phenotypes. Clinical studies confirm that loss-of-function variants in *KCNH2* are the primary cause of long QT syndrome type 2 (LQT2) and are significantly associated with epilepsy (particularly temporal lobe epilepsy) and elevated SUDEP risk (25, 36, 38). For instance, multiple *KCNH2* variants, including I82T, R863X, and R744X have been reported to concurrently cause LQTS and epilepsy phenotypes in the same families or patients (36, 38, 39). Notably, the scope of pathogenic variants extends to non-pore regions, with frameshift variants at the carboxyl terminus (e.g., P926AfsX14) also confirmed to cause LQT2 and *de novo* epilepsy (40). The common pathophysiological mechanism involves impaired channel function leading to delayed termination of electrical activity: prolonged action potential duration (APD) in the heart and neuronal hyperexcitability in the brain (37). A rabbit model carrying human *KCNH2* variants provides direct causal evidence for *KCNH2*-mediated cardio-cerebral comorbidity, recapitulating a composite phenotype of LQT2, spontaneous epilepsy, and sudden death. Studies show significantly prolonged QTc intervals in variant-carrying rabbits (*p* < 0.001), higher rates of seizure-like discharges (*p* < 0.003), and a sudden death rate rising dramatically from 1.5% in controls to 18.9% (*p* < 0.003), with death modes including seizure-mediated death and sudden cardiac death (35).

#### 3.1.2 *KCNQ1* (K_v_7.1)

Similarly, the *KCNQ1* gene, encoding the K_v_7.1 channel, provides further support for the concept of cardio-cerebral ion channelopathies. As the primary causative gene for long QT syndrome type 1 (LQT1), *KCNQ1*’s role is now recognized to extend beyond the myocardium. Multiple familial studies indicate that pathogenic *KCNQ1* variants (e.g., L273F, Q530X) can also cause epilepsy, establishing a direct genetic link between the heart and brain (41, 42). These studies reveal significant phenotypic heterogeneity, where family members carrying the same *KCNQ1* variant may exhibit only cardiac symptoms, neurological symptoms, or both (42). This phenomenon has important clinical implications: some LQTS patients with *KCNQ1* variants may initially present with seizure-like episodes, which are actually hypoxic events caused by cardiac polymorphic ventricular tachycardia (PMVT) (24). Such phenotypic overlap can easily lead to long-term misdiagnosis (24), delaying effective cardiac treatment and underscoring the necessity of systematic cardiac evaluation in patients with unexplained epilepsy.

#### 3.1.3 *SCN5A* (Na_v_1.5)

The *SCN5A* gene, encoding the voltage-gated sodium channel Na_v_1.5, is the third key gene linking cardio-cerebral comorbidity. A postmortem genetic analysis of SUDEP cohorts found that approximately 13% of cases carried LQTS-related gene variants (31). In SUDEP cases, multiple pathogenic *SCN5A* variants have been identified, such as the predicted deleterious *de novo* missense variant R523C (43). *SCN5A* variants are associated with various cardiac disorders, including long QT syndrome type 3 (LQT3) and Brugada syndrome. Given that Na_v_1.5 is also expressed in the central nervous system (particularly in the limbic system), this provides a biological basis for its involvement in neuronal function. In a specific family, the *SCN5A* W1095X truncating variant cosegregated with epilepsy and Brugada syndrome phenotypes, demonstrating that its dysfunction can simultaneously impair cardiac conduction and neuronal excitability, supporting the possibility that SCN5A dysfunction may contribute to combined neurocardiac vulnerability in selected families (32).

### 3.2 Other ion channels and signaling pathways

Beyond the core genes mentioned above, other ion channels and signaling pathways have been implicated in the molecular processes of cardio-cerebral comorbidity. For example, the *KCND3* gene, encoding the K_v_4.3 channel, has a V392I variant that caused a composite phenotype of early repolarization syndrome, paroxysmal atrial fibrillation, and epilepsy in one family (44). This variant confers a complex mixed electrophysiological phenotype on the K_v_4.3 channel, with heart rate-dependent function, which may explain its simultaneous induction of multiple cardio-cerebral symptoms. This discovery not only defines a new cardio-cerebral channelopathy but also highlights the need for systematic evaluation of non-classical arrhythmia genes and exploration of precision treatments (e.g., using quinidine) based on the electrophysiological characteristics of specific variants (45).

The molecular mechanisms of cardio-cerebral connections also involve intracellular calcium signaling pathways. Ryanodine receptor 2 (RyR2), the primary calcium release channel on the sarcoplasmic/endoplasmic reticulum, can lead to calcium “channel leak” when dysfunctional, which is the core pathology of catecholaminergic polymorphic ventricular tachycardia (CPVT). Studies found that gene knock-in mouse models carrying the CPVT-related *RyR2-R2474S* variant not only exhibit typical exercise-induced ventricular arrhythmias and sudden death but also display spontaneous epileptic seizures independent of cardiac events. The mechanism lies in the variant-bearing RyR2 channel exhibiting calcium leak in hippocampal neurons, leading to neuronal hyperexcitability. Importantly, the RyR2 stabilizer S107 not only prevents arrhythmias but also effectively raises the seizure threshold. This indicates that intracellular calcium homeostasis imbalance is another shared pathway linking cardiac electrical instability and neuronal hyperexcitability, providing a theoretical foundation for developing targeted drugs that simultaneously treat dual cardio-cerebral phenotypes (46).

### 3.3 Causal evidence from experimental models

Findings from human genetics studies have been validated in various experimental models, which provide critical causal evidence for understanding the pathophysiology of cardio-cerebral comorbidity.

Genetically engineered animal models have confirmed the pathological effects of shared ion channels. For example, heterozygous *Scn1a* variant mouse models modeling human Dravet syndrome not only exhibit severe epilepsy but also show increased sodium currents mediated by Na_v_1.5 in cardiomyocytes, prolonged action potential duration, and inducible arrhythmias (47). However, further research reveals more complex mechanisms: brain-specific (rather than cardiac-specific) Scn1a knockout is sufficient to recapitulate the SUDEP phenotype, with death closely associated with severe post-seizure bradycardia driven by brain-derived parasympathetic overexcitation (48). This suggests that, in addition to the direct effects of ion channels in the heart, the secondary influence of epileptic seizures on cardiac function via the autonomic nervous system is also a key pathway in SUDEP.

Furthermore, seizure-related physiological consequences can act as a “second hit,” exacerbating underlying cardiac electrical instability. In a canine LQT1 model, pharmacologically induced seizures directly trigger torsades de pointes (TdP), providing direct experimental evidence for this hypothesis (49). Research also reveals that epileptic activity can actively remodel cardiac electrophysiology. In chemically induced epilepsy rat models, sodium channel subtypes typically highly expressed in neurons (e.g., Na_v_1.1) are ectopically upregulated in cardiomyocytes, leading to significant increases in late sodium current (I_Na_L) and action potential prolongation. This pathological electrophysiological change is highly similar to the mechanism of LQT3, providing a molecular explanation for epilepsy-induced acquired cardiac electrical remodeling (50). Status epilepticus can also affect the heart through non-direct gene-mediated pathways, such as inducing cardiomyocyte hypoxia (marked by HIF-1α expression) and upregulating P-glycoprotein expression, resulting in QT interval prolongation and increased sudden death risk (51). These findings collectively indicate complex interactions between genetic susceptibility as an underlying substrate and seizure-related physiological consequences as acute triggers, which contribute to fatal cardiorespiratory events through multiple pathways, including direct ion channel dysfunction, brain-heart autonomic dysregulation, respiratory compromise, and secondary cardiac electrical remodeling.

## 4 The “second hit” hypothesis: central nervous system in cardiac electrophysiology

**Figure 2:**
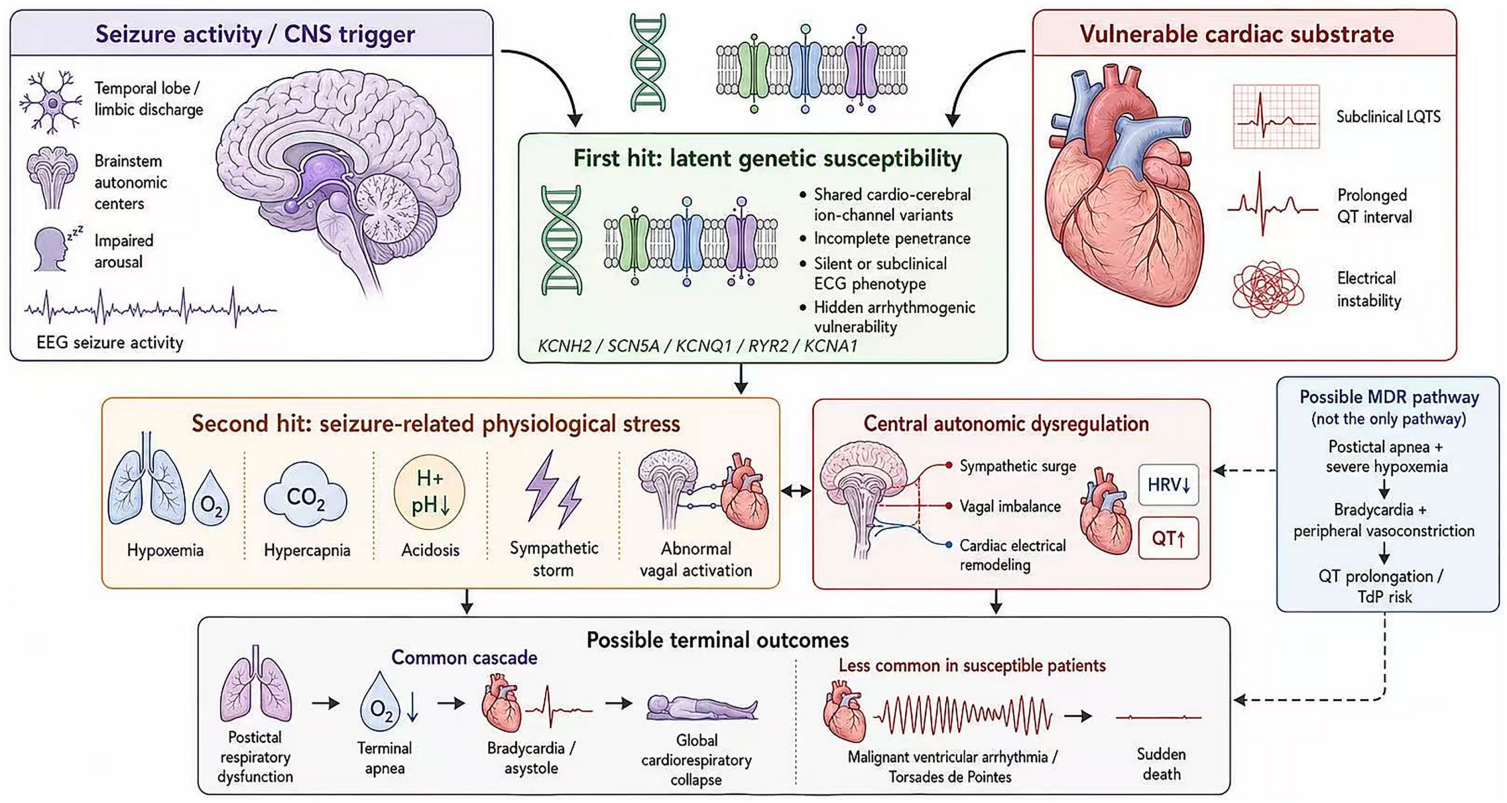
The two-hit pathophysiological model of sudden unexpected death in epilepsy (SUDEP). Latent genetic susceptibility in the form of shared cardio-cerebral ion-channel variants that cause subclinical LQTS or cardiac electrical instability constitutes the first hit. Postictal respiratory dysfunction, hypoxemia, hypercapnia, acidosis, sympathetic surge, and abnormal vagal activation are seizure-induced physiological stressors that act as the second hit by amplifying autonomic dysregulation and repolarization vulnerability. These interacting mechanisms may lead to QT prolongation, bradyarrhythmia or asystole, and terminal cardiorespiratory collapse. In susceptible individuals they may also trigger malignant ventricular arrhythmias such as torsades de pointes, albeit less frequently. MDR, mammalian diving reflex; SUDEP, sudden unexpected death in epilepsy; LQTS, long QT syndrome; TdP, torsades de pointes.

To clarify the pathophysiological mechanisms, we propose a refined two-hit framework distinguishing between the arrhythmogenic substrate and acute physiological triggers. In this model, genetic susceptibility (e.g., subclinical LQTS driven by shared cardio-cerebral gene variants) constitutes the underlying First Hit. Rather than the cortical electrical discharge itself acting as the second hit, the Second Hit comprises the downstream cardiorespiratory consequences of the epileptic seizure, such as severe hypoxemia, sympathetic storms, and acid–base imbalances. These seizure-related acute physiological stressors act as triggers, uncovering the hidden underlying genetic vulnerability and precipitating fatal arrhythmias (Figure 2).

### 4.1 Epilepsy-associated autonomic dysfunction and cardiac remodeling

Epilepsy exerts significant and often detrimental effects on cardiac function through central autonomic networks. Epileptic seizures, especially those originating in the limbic system, can induce severe autonomic dysfunction, characterized by reduced heart rate variability (HRV) and a profound autonomic imbalance from sympathetic overactivation and abnormal vagal tone (2, 52). This chronic, recurrent autonomic dysregulation can persistently remodel the cardiac electrophysiological substrate, increasing susceptibility to arrhythmias.

Even with normal cardiac ion channel function, CNS-originating specific channel defects can trigger cardiac events by disrupting central autonomic efferent pathways. For example, the *KCNA1* gene, encoding the K_v_1.1 potassium channel primarily expressed in neurons, leads to brain-derived cardiac dysfunction in mouse models when dysfunctional. The mechanism involves impaired vagal efferent signaling, resulting in cardiac conduction block and bradycardia, thereby increasing SUDEP risk (53). This study demonstrates that dysfunction of CNS-specific ion channels is sufficient to impair cardiac electrophysiological stability through neurogenic mediation.

Additionally, central regulation of the heart exhibits spatial specificity and hemispheric lateralization. Research shows that ictal cardiac repolarization changes in patients with mesial temporal lobe epilepsy are closely related to the side of seizure origin, with left hippocampal-origin seizures more likely to cause significant QT interval prolongation than right-sided ones (54). This provides evidence for fine, lateralized central control of the heart and suggests that epileptic activity in specific brain regions (e.g., left temporal lobe) may be a high-risk factor for inducing arrhythmias.

### 4.2 Epileptic seizures as acute triggers for cardiac events

The acute physiological effects of epileptic seizures, particularly postictal respiratory suppression and severe hypoxemia, can activate the mammalian diving reflex (MDR). MDR simultaneously induces bradycardia and peripheral vasoconstriction. In individuals with underlying cardiac repolarization defects, this reflex further exacerbates bradycardia and QT interval prolongation, creating conditions for torsades de pointes (TdP) (55). Thus, MDR triggered by postictal respiratory dysfunction may represent one potential pathway linking epilepsy, LQTS, and sudden death in susceptible individuals.

Respiratory dysfunction should be considered a core component of SUDEP pathophysiology, not merely an upstream trigger for arrhythmia. GTCS may produce central apnea, hypoventilation, oxygen desaturation, hypercapnia, impaired arousal, airway obstruction, and brainstem-mediated cardiorespiratory collapse (4, 56–58). Hypoxemia and hypercapnia can then aggravate autonomic instability and myocardial electrical vulnerability.

MORTEMUS showed that monitored SUDEP and near-SUDEP often followed a recognizable sequence: rapid breathing after GTCS, transient or terminal cardiorespiratory dysfunction within minutes, later terminal apnea, and then cardiac arrest (56). Importantly, terminal apnea was commonly observed before terminal asystole in monitored cases, which argues against presenting primary ventricular tachyarrhythmia as the usual terminal event. Non-seizure SUDEP cases further demonstrate that profound EEG suppression with respiratory and bradyarrhythmic dysfunction may occur even without an immediately observed electroclinical seizure, emphasizing heterogeneity (57).

The mammalian diving reflex may still be relevant. Postictal apnea and severe hypoxemia can trigger bradycardia and peripheral vasoconstriction. In a patient with repolarization vulnerability, this may further prolong QT and lower the threshold for malignant arrhythmia (55). However, this mechanism should be framed as one possible interaction between respiratory and cardiac stress rather than the dominant pathway in all SUDEP cases.

Seizures can generate phase-dependent autonomic imbalance, typically a sympathetic surge followed by abnormal vagal activation, which together may precipitate bradycardia/asystole in susceptible individuals.

Brain structural lesions themselves are also important risk modulators. Clinical studies show that patients with structural brain diseases (e.g., stroke, tumors) exhibit more pronounced QT interval prolongation post-seizure compared to those without clear structural abnormalities (59). This suggests that abnormalities in brain structure and function may disrupt normal autonomic regulatory networks, lowering the cardiac tolerance threshold under the physiological stress of epileptic seizures.

### 4.3 Integrative “two-hit” model for SUDEP

Synthesizing the above evidence, an integrative “two-hit” hypothesis incorporating genetic susceptibility and physiological stress has been proposed to explain SUDEP mechanisms. This hypothesis posits that the first hit is the patient’s genetic susceptibility, such as an incompletely penetrant or mildly pathogenic cardiac ion channel gene variant, which may manifest only as subclinical ECG abnormalities or even appear normal at rest (39).

The second hit comprises seizure-related acute physiological stressors, including postictal respiratory dysfunction, severe hypoxemia, hypercapnia, autonomic storms, acidosis, and electrolyte disturbances. In this setting, the arrhythmogenic potential of the originally “silent” genetic defect is dramatically amplified and exposed, ultimately precipitating fatal cardiorespiratory collapse, which may include bradyarrhythmia, asystole, or, less commonly, malignant ventricular arrhythmias in susceptible individuals (39). This model not only reasonably explains the substantial phenotypic variability among individuals carrying the same gene variants but also clarifies the central triggering role of epileptic seizures in converting latent genetic risks into actual sudden death events.

### 4.4 Limitations of the proposed two-hit model

The proposed two-hit model is a heuristic framework, not a universal explanation for SUDEP. SUDEP is mechanistically heterogeneous, and available monitored cases more often support a cascade involving postictal respiratory dysfunction, impaired arousal, autonomic disturbance, bradycardia, and terminal cardiorespiratory collapse than a single primary ventricular tachyarrhythmia pathway (56, 57). Most human genetic evidence remains associative, and prospective population-level data showing primary ventricular arrhythmia as a common terminal SUDEP mechanism are limited (18, 19, 39). For these reasons, the model is best applied to selected high-risk patients in whom genetic, ECG, family-history, seizure, and respiratory features suggest combined neurocardiac vulnerability.

## 5 Diagnosis, risk stratification, and treatment from an integrative perspective

The integrative framework of “cardio-cerebral ion channelopathies” offers direct guidance for clinical practice, particularly in diagnosis, risk stratification, and treatment decisions.

### 5.1 Diagnostic considerations and integrative screening

The significant phenotypic overlap between SUDEP and LQTS poses challenges to accurate diagnosis and may lead to inappropriate clinical management. For example, cardiac syncope in LQTS patients is often accompanied by convulsive activity, easily misdiagnosed as primary epilepsy, resulting in unnecessary anti-seizure medication (ASM) therapy while the fatal cardiac risk remains unidentified (23, 24, 26). Therefore, baseline or repeated ECG assessment should be considered, particularly in patients with unexplained syncope, seizure-like episodes with atypical features, suspected drug-resistant epilepsy, abnormal cardiac symptoms, or a family history of LQTS or sudden death. In clinical practice, rather than advocating for blanket arrhythmia-gene screening, we envisage a prioritized, targeted genetic screening approach restricted to selected high-risk epilepsy subgroups (e.g., individuals with a confirmed family history of LQTS, unexplained sudden cardiac death, or persistent unexplainable QTc prolongation on serial ECGs). However, it is critical to acknowledge that due to the subclinical and incomplete penetrance nature of LQTS, many at-risk individuals may remain asymptomatic and may not be readily identifiable using conventional clinical risk markers alone, posing a significant challenge to targeted risk stratification.

Conversely, in diagnosed LQTS patients, potential neurological comorbidities should be considered. Some LQTS patients exhibit nonspecific electroencephalogram (EEG) abnormalities, suggesting possible primary cortical dysfunction (20, 28). Thus, systematic neurological evaluation is necessary for high-risk LQTS patients. Additionally, while genetic testing is increasingly utilized in research, it is not currently standard care for routine arrhythmia screening in all epilepsy patients. Given the challenges in interpreting the pathogenicity of shared gene variants such as *KCNQ1*, *KCNH2*, and *SCN5A*, clinicians must avoid over-translating these findings. Variant incomplete penetrance, broad phenotypic spectra, and numerous variants of uncertain significance (VUS) necessitate ongoing functional validation and cautious evidence assessment before integrating such data into clinical decision-making (45).

### 5.2 Cardiac effects of anti-seizure medications

Under the framework of cardio-cerebral ion channelopathies, selecting ASMs must involve assessing their potential impact on cardiac electrophysiology. Certain ASMs, particularly sodium channel blockers (e.g., lamotrigine, phenytoin), may prolong the QT interval by directly inhibiting hERG potassium channels or affecting sodium channel function, thereby increasing cardiac event risk in specific LQTS genotypes (60, 61). Concurrently, drug metabolic interactions may also lead to QT interval prolongation or Brugada-like ECG changes, elevating the risk of cardiac arrest (62).

Precision medicine approaches are key to addressing this challenge. Research indicates that sodium channel blockers have differential effects across LQTS genotypes, increasing risk in LQT2 patients while potentially protecting LQT1 patients (60). This finding provides evidence for genotype-based personalized medication strategies and suggests that genotype-guided ASM selection is an emerging, hypothesis-driven approach rather than current standard care. While potentially valuable for high-risk cohorts, evidence does not yet support routine arrhythmia gene screening in all epilepsy patients. Future implementation of this strategy must be cautious to avoid the over-translation of preliminary genetic and pharmacological data into current clinical practice (63). To clarify, this framework does not imply that routine or universal arrhythmia-gene screening is indicated prior to the initiation of ASM therapy, which would not reflect current standard clinical practice. Instead, clinicians should rely on standard cardiological evaluations (such as baseline ECGs) for the vast majority of patients, reserving genetic testing only for the strictly defined high-risk subgroups mentioned above.

## 6 Conclusions and future perspectives

SUDEP and LQTS, while often clinically distinct, may in some cases reflect a risk-modifying spectrum of “cardio-cerebral ion channelopathies.” Rather than a universal model, this framework highlights that shared ion channel dysfunction can, in certain predisposed individuals, contribute to overlapping pathologies. A central viewpoint of this review is that, in selected patients, the interaction between genetic susceptibility and seizure-induced physiological stress may represent an important risk-modifying mechanism linking epilepsy, LQTS, and sudden death vulnerability. This integrative model provides a theoretical framework for explaining their clinical overlap and genetic associations. However, despite growing recognition of static genetic risks, there is currently a lack of effective biomarkers that integrate multimodal physiological data for dynamic prediction of individual sudden death risk. The integrative concept of “cardio-cerebral ion channelopathies” opens new avenues for drug development. Future therapeutic strategies should transcend symptomatic treatment, exploring drugs that simultaneously stabilize neuronal and cardiomyocyte membrane potentials. For example, novel potassium channel openers or *RyR2* stabilizers may, in selected genotypic contexts, offer dual neuroprotective and cardioprotective potential by reducing neuronal hyperexcitability while mitigating cardiac electrical instability (37, 46).

Future therapeutics may benefit from precision medicine approaches developed in inherited arrhythmia syndromes. In LQTS, genotype-specific strategies include late sodium current blockade for LQT3, efforts to enhance I_Ks_ or restore I_Kr_ function, hiPSC-derived cardiomyocyte platforms for variant-specific drug testing, and experimental gene replacement, gene silencing, or suppression-and-replacement approaches (64). For RyR2-related disorders, contemporary work distinguishes gain-of-function and loss-of-function ryanodinopathies and highlights RyR2-directed pharmacology, flecainide, quinidine in selected models, and emerging RyR2 modulators as areas of investigation (65).

Potassium channel openers also represent a relevant class of mechanism-informed therapies. Retigabine/ezogabine provided clinical proof that positive modulation of neuronal K_v_7 channels can reduce excitability, although its use was limited by safety and tolerability concerns (66). Newer K_v_7 modulators, such as XEN1101, have renewed interest in this therapeutic class for focal epilepsy (67). However, whether K_v_7 modulation can reduce SUDEP risk remains unknown, and future studies would need to evaluate seizure reduction together with respiratory, autonomic, and cardiac safety endpoints.

For epilepsy and SUDEP, these strategies remain investigational. Translation will require genotype-specific functional validation, prospective neurocardiac cohorts, synchronized EEG-ECG-respiratory monitoring, standardized SUDEP phenotyping, and careful distinction between association and causation. A realistic goal is not universal genetic screening or universal genotype-based prescribing, but better identification of selected patients in whom cardiac substrate, respiratory vulnerability, seizure burden, and medication exposure combine to increase danger. Prospective, genotype-enriched neurocardiac cohorts with synchronized EEG-ECG-respiratory monitoring and prespecified autonomic/respiratory endpoints will be essential to determine when and how neurocardiac substrates modify SUDEP risk.
